# Supplementary material for: Long-term impact of 10-valent pneumococcal conjugate vaccine among children <5 years, Uganda, 2014–2021
Source: PLOS Glob Public Health. 2025 Jan 6;5(1):e0002980. doi: 10.1371/journal.pgph.0002980 (PMC11703042; doi:10.1371/journal.pgph.0002980)
Supplement: Attachment — Submitted filename: review-Sarashwati Giri-Uganda study.docx [file pgph.0002980.s001.docx]

# Background of the study

Clarity and Focus: Streamline the material to highlight essential elements pertinent to the study's aims. Avoid going into excessive detail about other issues or material that isn't immediately relevant to the study's objective.

Scope and Context: Emphasise how therapies like PCV10 are closely related to the study's progress evaluation. Concentrate on how these treatments affect pneumonia admissions and death among Ugandan children under the age of five.

Structure and Flow: Organise the background information such that it flows logically from global concerns to the specific situation in Uganda. This will assist to better contextualise the study's aims.

# Methodology

Data Quality: Consider providing more detail on the quality and reliability of the data collected from the District Health Information System version 2 (DHIS2) to strengthen the study's credibility.

Methodological Clarity: The methodology is comprehensive but could benefit from a clearer explanation of how the specific data was extracted and verified for accuracy.

Geographical Coverage: The study examines national and regional data; ensure that this distinction is clearly stated throughout the methodology to avoid confusion.

Data Analysis: More information on the statistical methods used (e.g., thresholds for significance in the Mann-Kendall test) could improve clarity.

Ethical Considerations: The ethical section is thorough, but consider adding any additional safeguards in place to protect the data and its usage.

Overall, the methodology is thorough, but providing more details on data quality assurance and specific analytical methods could enhance clarity and robustness.

**Results**

- The results section is mostly clear and presents key findings well. However, consider the following for potential improvements:
- Context: Provide more context on possible confounding factors that may impact trends.
- Maps: Ensure visual data (maps) are clearly referenced and easy to interpret.
- Statistical Analysis: Clarify statistical methods and their interpretation for better understanding.

**Discussion**

The discussion provides a comprehensive analysis of the trends in pneumonia hospital admissions, mortality, and case-fatality among children under 5 in Uganda from 2013 to 2021. Here are some suggestions for improvement:

Clarification: Further clarify the specific interventions, such as the introduction of PCV10 and the WHO-recommended admission criteria, and how these influenced the observed trends.

Comparative Analysis: Provide a more direct comparison with other African countries' data to highlight differences and similarities in pneumonia trends.

Regional Differences: Explore the reasons behind regional variations in pneumonia cases and deaths, offering potential explanations or hypotheses for further research.

Limitations: Ensure limitations are concise and directly address potential biases or areas for improvement in the study.

Further Recommendations: Suggest additional research avenues or data collection methods that could provide more insight into the study’s findings.
