# Supplementary material for: Long-term impact of 10-valent pneumococcal conjugate vaccine among children <5 years, Uganda, 2014–2021
Source: PLOS Glob Public Health. 2025 Jan 6;5(1):e0002980. doi: 10.1371/journal.pgph.0002980 (PMC11703042; doi:10.1371/journal.pgph.0002980)
Supplement: Attachment — Submitted filename: Reviewer Comments.docx [file pgph.0002980.s002.docx]

We greatly appreciate the comments and suggestions made by the authors. Here is a point by point response to the comments and suggestions

Reviewer #1:

**Comment 1** Background: Clarity and Focus: Streamline the background to emphasize the study's aims and its relevance to PCV10 and pneumonia outcomes among children under 5 in Uganda.

**Response 1:** Revisions made to emphasize aims of the emphasize the study's aims and its relevance to PCV10 and pneumonia outcomes among children under 5 in Uganda.

**Comment 2:** Scope and Context: Focus on the relationship between therapies like PCV10 and the study's objectives, particularly regarding pneumonia admissions and mortality.

**Response 2:** Revisions made to focus on relationship between PCV10 and pneumonia admission and mortality

Line 70-72:… PCV contains serotypes that are commonly associated with invasive and mucosal pneumonia among children <5 years and can reduce both incidence and severity of disease leading to reductions in admissions and mortality^4^.

**Comment 3** Structure and Flow: Organize the background to logically flow from global concerns to the specific context of Uganda.

**Response 3:** Revisions made in paragraph one for the background to flow from global to Ugandan context.

**Methodology:**
**Comment 4:** Data Quality: Provide more details on data quality and reliability from DHIS2 to bolster credibility.

**Response 4:** Data quality details have been added line 109 to 111

…To ensure data quality within DHIS2,data in physical health registers are routinely compared with summary reports within the electronic system in addition to the in-built quality checks^15^

**Comment 5:** Methodological Clarity: Offer clearer explanations of data extraction, verification, and analytical methods.

**Response:** data extraction, verification and analytical methods revised as follows: Line 138 to line 148

We downloaded data from the DHIS2 platform as in Excel file and imported it into EpiInfo 7 software (CDC, Atlanta, USA) for analysis. Annual proportion of pneumonia cases admitted, mortality rates, and case-fatality rates were calculated at national and regional levels. Line graphs were used to describe national and regional trends. The Mann-Kendall test was conducted to assess trends in annual proportion of pneumonia cases admitted, mortality rates, and case-fatality rates. The Z value >0 and P<0.05 indicated an increased trend while the Z value >0 and P<0.05 represented a decreased trend. Choropleth maps drawn using QGIS software were used to show the spatial distribution of pneumonia admissions and mortality in the country.

**Comment 6** Geographical Coverage: Clearly distinguish between national and regional data to avoid confusion.

**Response 6:** National and regional distinctions added to titles of each section

**Comment 7** Data Analysis: Clarify the statistical methods used, including significance thresholds.

**Response 7:** Data analysis methods have been clarified.

Line 138 to line 148

We downloaded data from the DHIS2 platform as in Excel file and imported it into EpiInfo 7 software (CDC, Atlanta, USA) for analysis. Annual proportion of pneumonia cases admitted, mortality rates, and case-fatality rates were calculated at national and regional levels. Line graphs were used to describe national and regional trends. The Mann-Kendall test was conducted to assess trends in annual proportion of pneumonia cases admitted, mortality rates, and case-fatality rates. The Z value >0 and P<0.05 indicated an increased trend while the Z value >0 and P<0.05 represented a decreased trend. Choropleth maps drawn using QGIS software were used to show the spatial distribution of pneumonia admissions and mortality in the country.

**Comment 8**: Ethical Considerations: Ensure the ethical considerations are thorough and highlight data protection measures.

**Response 8:** Ethical statement revised as follows

This study was done to inform public heath practice and therefore determined as non-research and therefore waived of the full institutional review board. The Ministry of Health (MoH) of Uganda through the office of the Director General Health Services gave approval to access data from the national electronic surveillance data base DHIS2. Our study utilized routinely generated aggregated surveillance data with no personal identifiers in health facility in-patient monthly reports through the DHIS2. We stored the abstracted data set in a password-protected computer and only shared it with the investigation team.This activity was reviewed by CDC and was conducted consistent with applicable federal law and CDC policy.§ §See e.g., 45 C.F.R. part 46, 21 C.F.R. part 56; 42 U.S.C. §241(d); 5 U.S.C. §552a; 44 U.S.C. §3501 et seq
**Results:**

**Comment 9 Context**: Offer context on possible confounding factors affecting trends.

Response: Context on possible counfounders was not assessed in the study and has therefore been discussed in the methods section. Line 213-218

**Comment 9** Maps: Ensure visual data (e.g., maps) is clear and easy to interpret.

**Response**: Maps have been clarified to ease interpretation.

**Comment 11Statistical** Analysis: Clarify statistical methods and their interpretation for better comprehension.

**Response:** Statistical methods clarified. Line 128 to line 132

**Discussion:**
**Comment clarification 12**: Further clarify the impact of specific interventions on the observed trends.

**Response:** We discuss trends in context of interventions (mainly PCV10 coverage) to address potential impact. Due to limitations of the study design, we are unable to assess the impact of specific interventions.

**Comment Comparative Analysis 13:** Directly compare findings with other African countries to highlight differences and similarities.

**Response:** We compare percentage declines documented Burkina Faso, Zambia, South Africa and Rwanda and explore possible reasons for differences.

**Comment Differences 14:** Explore reasons for regional variations in pneumonia outcomes, providing hypotheses for further research.

**Response:** We have explored poverty and housing as a possible reason for variations in case fatality rates and mortality. Line 239-240.Since little is documented in this aspect we recommend further studies

**Comment Limitations 15:** Present limitations succinctly and address potential biases or areas for improvement.

**Response:** We have revised limitations and potential biases. Line 244- 255
**Comment Further Recommendations 16:** Suggest additional research avenues or data collection methods for deeper insight.
By incorporating these suggestions, the study can enhance its clarity, robustness, and overall contribution to understanding pneumonia outcomes in Uganda.

**Response:** This has been revised as follows. Line 265 to 267 . …..We recommend future studies monitoring of trends in the circulating pneumococcal serotypes in the post-PCV.

Reviewer #2: The study is robust, relevant and contributes to the body of scientific evidence. The authors studied the long term impact of PCV10 among under five children in Uganda using a national surveillance secondary data.

**Comments 17**
The title does not fully project the findings and significance of the study. Indeed a search for the purpose of systematic review regarding PCV impact could easily miss this paper when published.
I would suggest you re-craft the title to show at a glance that this paper is aimed at evaluating the long term impact impact of PCV10 among Ugandan under five children.

**Response 17:** Title revised to read as follows: Long-term impact of 10-valent pneumococcal conjugate vaccine among children <5 years, Uganda, 2014–2021.

**Comment 18 :**The authors mentioned ages for PCV vaccination as 6, 10 and 12 weeks; is this correct or did they mean 6, 10 and 14 weeks? Doses are given at least weeks apart or is Uganda giving third dose after 2 weeks?

**Response 18**: This has been corrected to 14 weeks

**Comment 19:** The authors noted that the official rollout of PCV10 was in 2014, would it not be better to align analysis and title to 2014 instead of 2013 for uniformity?
Line 131 - "Although the PCV10 vaccine was introduced in 2013, official rollout to the entire country started in 201417. PCV 3 vaccine coverage was therefore calculated from 2014 to 2021." Everything should be calculated from 2014.

It is unclear if authors are assessing 2013-2021 or 2014-2021. When they say 9years, the period is not clear as the months in 2013/2014 and 2021 included in the study are unclear.
I suggest mentioning the months and limiting the entire analysis to 2014-2021, rather analysing coverage from 2014 and other analyses from 2013, and possibly reflecting this on the title.

**Response 19:** Revision made to state analysis period as January 2014 to December 2021.

The authors did not say anything about indirect effects. Would have been interesting to measure indirect effects on older age groups? This further amplifies the impact of PCV10 as has been documented elsewhere.

Comment Discussion
Lines 205-207 - Generally good discussion on possible confounders especially of revised WHO admission guidelines, but many impact studies have shown positive results and Uganda may not be an exception especially with the high vaccination coverage and national data.

Comment Line 226-235 - Were the cited studies on Vaccine strain replacement in u5 or adults?
There is need for further studies on serotypes and trends in older children and adults in Uganda.
**Response**: Cited studies are on serotype replacement among under 5s
 **Comment** :Figures
Merge figs 1-3 as 1a-c
Merge figs 4-5 as fig 2a-b

Response Figures have been merged
